# Supplementary material for: Compressed representation of brain genetic transcription
Source: Hum Brain Mapp. 2024 Jul 24;45(11):e26795. doi: 10.1002/hbm.26795 (PMC11267301; doi:10.1002/hbm.26795)
Supplement: Supplementary file 1 — Figure S1. Histograms of transcriptomic data at 4 mm3 (A) and 8 mm3 resolutions (B) shows the distribution to be Gaussian. Heatmaps in (C) and (D) show individual voxel x gene expression values in 4 and 8 mm3 resolutions, respectively. [file HBM-45-e26795-s001.pdf]

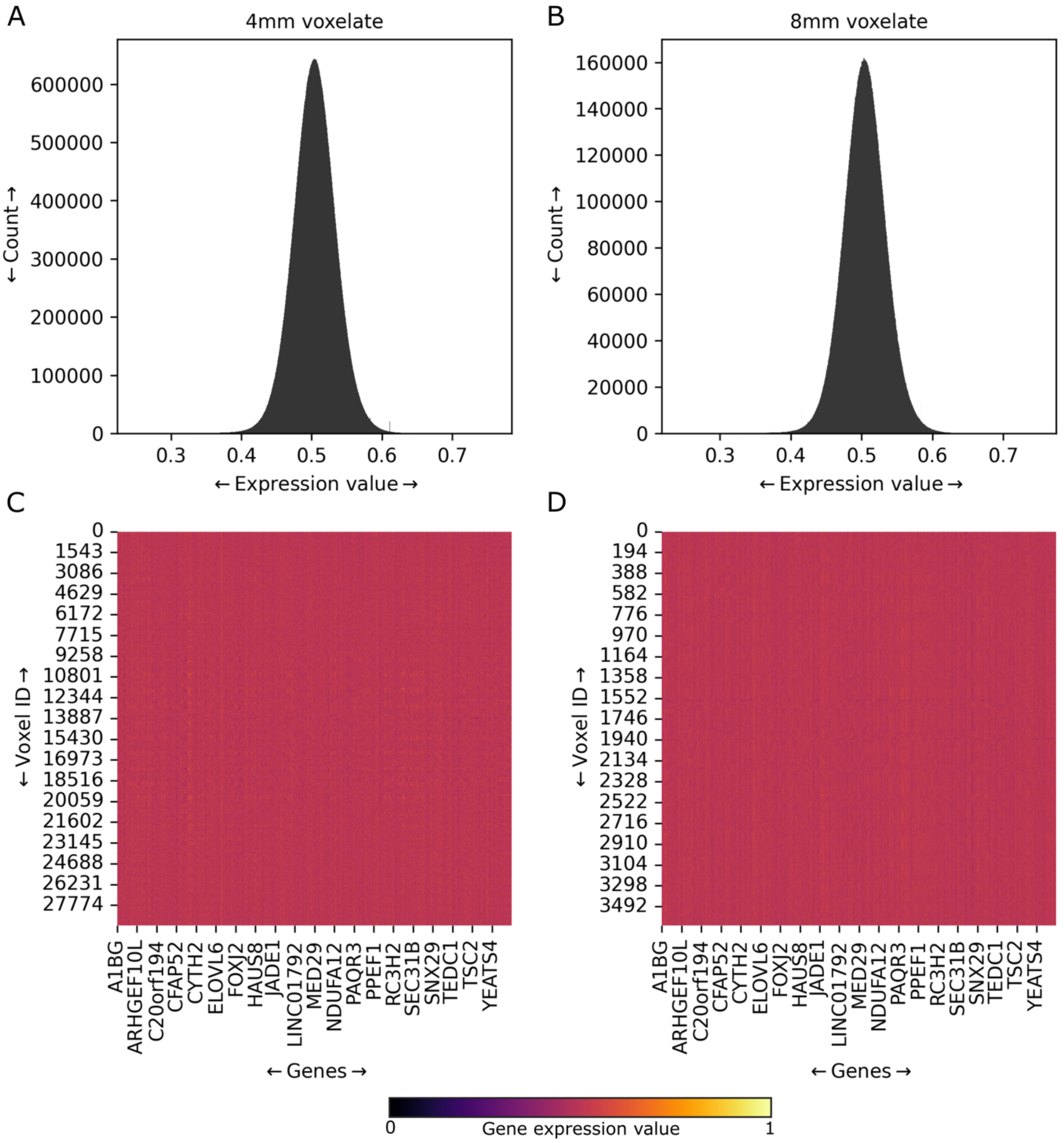

Supplementary Figure 1. Histograms of transcriptomic data at 4mm^3 (A) and 8mm^3 resolutions (B) shows the distribution to be Gaussian. Heatmaps in C) and D) show individual voxel x gene expression values in 4mm^3 and 8mm^3 resolutions, respectively.
